# Follicular Variant of Acquired Dermal Macular Hyperpigmentation: A Case Report

**DOI:** 10.7759/cureus.34133

**Published:** 2023-01-24

**Authors:** Ammar A Bakhsh, Basant A Alzubaidy, Mohammed A Attas, Khalil F Miyajan, Khalid Al Hawsawi

**Affiliations:** 1 Faculty of Medicine, Umm Al Qura University, Makkah, SAU; 2 Dermatology, King Abdulaziz Hospital, Makkah, SAU

**Keywords:** admh, lichen planopilaris (lpp), lichen planus pigmentosus, follicular lichen planus pigmentosus, acquired dermal macular hyperpigmentation

## Abstract

Acquired dermal macular hyperpigmentation (ADMH) is a term used to describe a group of diseases that are characterized by idiopathic macular dermal hypermelanosis. These skin conditions include erythema dyschromicum perstans, lichen planus pigmentosus, and pigmented contact dermatitis, also known as Riehl's melanosis. This case report involves a 55-year-old woman who was generally healthy but who had been experiencing asymptomatic, slowly progressive skin lesions for the previous four years. A thorough inspection of her skin revealed many non-scaly, pin-point follicular brown macules, which in some spots had coalesced into patches across her neck, chest, upper extremities, and back. Darier disease and Dowling-Degos disease were included in the differential diagnosis. The biopsies of the skin revealed follicular plugging. The dermis had pigment incontinence with melanophages and slight perivascular and perifollicular mononuclear cell infiltrates. The patient was diagnosed with a follicular form of ADMH. Patient's skin condition caused her concern. She was reassured and prescribed topical steroids 0.1% betamethasone valerate ointment application twice a day for two days per week (weekends) and 0.1% tacrolimus ointment application twice a day for five days per week for three months. She showed some improvement and was put under periodic follow-ups.

## Introduction

Acquired dermal macular hyperpigmentation (ADMH) refers to a category of dermatoses best described by idiopathic macular dermal hypermelanosis. These include erythema dyschromicum perstans (EDP), lichen planus pigmentosus (LPP), and pigmented contact dermatitis known as Riehl's melanosis. Both clinically and histopathologically, these entities have a lot in common with one another. Clinically, they present as asymptomatic macules and patches that vary in color from gray to brown [1]. On a histopathological level, they are characterized by signs of current or resolved interface dermatitis, including pigment incontinence, vacuolar degeneration of the basal layer, and lichenoid tissue response. In contrast to LPP, follicular LPP (FLPP) manifests first as slate-gray follicular macules and typically appears during times of disease instability. It mostly affects the upper limbs and trunk of the body. It also has a female preponderance; however, it occurs in a younger age compared to most other types [2].

## Case presentation

**Figure 1:**
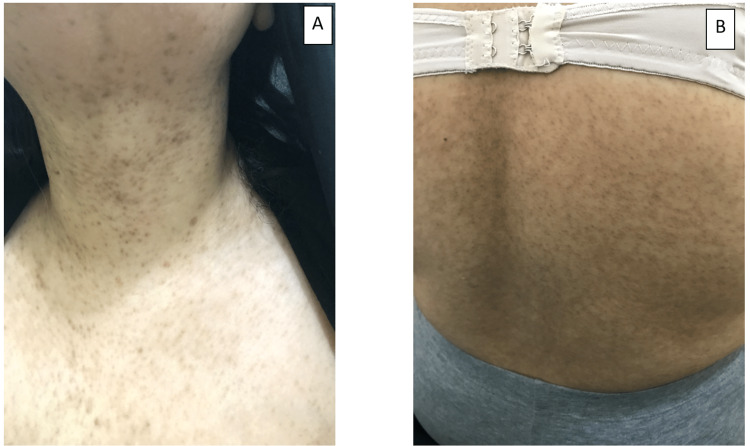
Multiple non-scaly, pin-point follicular brown macules coalescing in some areas into patches on patient's (A) neck and (B) back

A 55-year-old woman, otherwise healthy, presented with a four-year history of asymptomatic, slowly progressing skin lesions. There was no family history of these lesions, and the parents were not consanguineous. Her systemic examination was unremarkable. Her skin examination revealed multiple non-scaly, pin-point follicular brown macules, coalescing in some areas into patches over her neck, chest, upper extremities, and back (Figure 1). Hair, nails and mucus membrane examinations were all normal.

**Figure 2:**
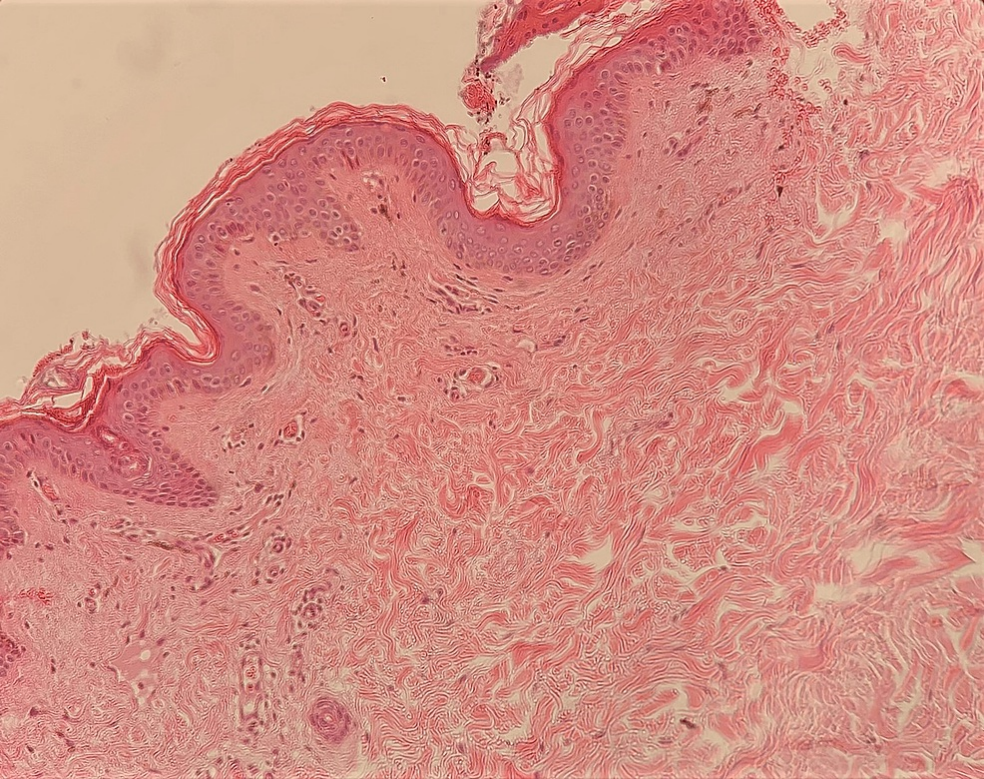
Skin biopsy from a brown patch from the neck The epidermis showed follicular plugging. The dermis showed pigment incontinence with melanophages, and mild perivascular and perifollicular mononuclear cellular infiltrates.

The main differential diagnosis included Darier disease and Dowling-Degos disease (DDD). A skin biopsy was taken from a brown patch on her neck. It showed follicular plugging, and the dermis showed pigment incontinence with melanophages, and mild perivascular and perifollicular mononuclear cellular infiltrates (Figure 2).

Based on previous clinico-pathological findings, the patient was diagnosed with the follicular variant of ADMH. She was concerned because of her skin disease, and hence was reassured. She was prescribed topical steroids 0.1% betamethasone valerate ointment application twice a day for two days per week (weekends) and 0.1% tacrolimus ointment application twice a day for five days per week for three months, with some improvement shown. She was also put under periodic follow-ups.

## Discussion

The term “ADMH”, which includes Riehl's melanosis, EDP, and LPP, was coined recently. Experts have unified these diseases under the label ADMH due to the great degree of clinical and histological similarities among these three entities as well as the occurrence of overlaps in certain instances. Clinically, they present with gray-brown macules and patches that are asymptomatic. LPP is usually located on sun-exposed areas and flexures, whereas EDP is usually located on sun-protected areas. Riehl’s melanosis favors sites with previous contacts with irritants. They are distinguished histopathologically by melanophages, basal layer vacuolar degeneration, and lichenoid tissue responses [1,3]. Dermoscopic features of ADMH include pigment dots, globules, and blotches. These features represent the lichenoid tissue damage associated with melanin incontinence. Other dermoscopic features include owl‑eye‑like structures that represent follicular clogging, and an enlarged pseudoreticular pattern, all of which correlate to an elevated melanin concentration in basal keratinocytes [4].

The etiology of these three entities is unknown. However, mustard oil, cosmetics, amla oil, and hair dyes have been implicated as etiological factors in LPP. Radiographic contrast agents, intestinal whipworm infection, ammonium nitrite, and cobalt allergy have also been implicated as etiological factors in EDP. Antigens present in cosmetics and textiles, especially para‑phenylenediamine, have been reported as etiological factors for Riehl’s melanosis [1].

The main differential diagnosis in our case includes Darier disease and DDD; however, clinically, Darier disease is characterized by follicular papules rather than macules. In DDD, the hyperpigmented lesions are maculo-papular, reticular and they involve the flexures in addition to histological characteristics. Darier disease, DDD and FLPP, all have peculiar histopathological features [2,3,5].

In the literature search, we found no mention of a follicular variant of EDP; instead there were several reports of FLPP. We think that the term follicular ADMH would be a better term. LPP has been reported in many forms, including diffuse, reticular, blotchy, follicular, perifollicular, inverse, mucosal, linear, and zosteriform patterns [2,5,6]. In the perifollicular variant of LPP (PLPP), the lesions involve the area surrounding the hair follicle. PLPP does not occur during disease instability [2,6]. In two cases, FLPP and lichen plano-pilaris of the scalp were both present, suggesting that there is a possibility that FLPP is a macular form of lichen plano-pilaris [2].

In a study, Sindhura et al. reported six cases of FLPP where the patients had asymptomatic pin-point slate-gray macules over the trunk, forearms, and hands (three patients) as well as across the dorsa (three patients). All six patients who underwent skin biopsy from follicular macules showed follicular plugging, localized basal cell vacuolization, elevated basal layer melanin, and a moderate to dense, primarily perifollicular lymphohistiocytic infiltration in the dermis [2]. In comparison to previously reported cases, our case showed a similar clinical pattern. However, the skin biopsy results in our case showed follicular plugging, pigment incontinence, and melanophages in the upper dermis, and mild perivascular and perifollicular mononuclear cellular infiltrates in the dermis. The pigment incontinence and melanophages in the upper dermis are considered evidence of resolved interface dermatitis.

The treatment of ADMH can be difficult. Given the ineffectiveness of various treatment modalities across the ADMH spectrum, cosmetic camouflage should be considered. The treatment of LPP that has been reported in the literature includes the avoidance of exacerbating factors, photoprotection, topical steroids, topical tacrolimus, topical antioxidants, low‑fluence Q‑switched Nd‑YAG, systemic corticosteroids, dexamethasone 2.5-5 mg orally administered twice weekly, dapsone, colchicine, isotretinoin, and mycophenolate mofetil. The treatment of EDP that has been reported in the literature includes griseofulvin and dithiazanine iodide, an anthelmintic drug, and clofazimine. The treatment of Riehl’s melanosis that has been reported in the literature includes the avoidance of the suspected agent; for example, the avoidance of hair dye/henna is crucial for its management. Hydroquinone, tretinoin, and glycolic acid-containing topical bleaching treatments have been used, but the results have been inconclusive. Oral tranexamic acid and low‑pulse energy 1064‑nm Q‑switched Nd‑YAG lasers have shown good efficacy [1,2,5]. In our case, the patient was prescribed topical steroids 0.1% betamethasone ointment and 0.1% tacrolimus ointment for three months, with some improvement shown. She was also put under periodic follow-ups, and is still followed up.

## Conclusions

ADMH is an umbrella term that is used to describe Riehl's melanosis, EDP, and LPP. The follicular variant of ADMH is among the very few conditions characterized by pin-point follicular pigmented macules. Darier disease is the principal differential diagnosis of pin-point follicular pigmented macules. It is characterised by follicular papules rather than macules. In DDD, the flexures are affected by maculo-papular, reticular hyperpigmented lesions. Darier disease, DDD, and the follicular variant of ADMH exhibit unique histopathological features.
